# The Relation between Vitality and Bulk

**Published:** 1908-10-03

**Authors:** 


### The Hospital

A Journal of

The Medical Sciences and Hospital Administration.

New Series. No. 82, Vol. IV. [No. 1154, Vol. XLV.] SATURDAY, OCTOBER 3, 1908.

### The Relation between Vitality and Bulk.

It has long been an accepted principle of life assurance that pronounced departures from the average ratio between height and weight at any given age are inimical to longevity; and those who present such anomalies are being daily called upon to pay extra premiums solely on this account, and to their own great indignation. In this, as in a number of other points where medicine and life assurance meet, the practice of life offices is a fine corrective to theorising: for this practice is founded on cold commercial considerations, and any theory which purports to explain it, yet fails to establish its commercial importance, has but a short shrift. In a recent communication to the Medical Society of New Jersey Dr. Brandreth Symonds, chief medical director of the New York Mutual Life Office, deals at some length with the experience of his office in the matter of overweights and underweights, and since longevity is a question of general importance in medicine, his figures deserve the attention of the profession at large. For life assurance purposes an individual is not considered an overweight unless his weight is more than 20 per cent. above the average for his height and age. It will be seen, therefore, that considerable provision is made for what may be called normal variations. But once the 20 per cent. excess is passed the disproportion becomes definitely unfavourable to length of life. The degree of danger is determined by two factors—namely, the extent of the excess and the age of the individual. For example, a man 40 years of age and 5 ft. 6 in. in height should weigh 150 lb. At 180 lb. he is 20 per cent. in excess, and at 195 lb. he is 30 per cent. in excess. Experience shows that a large group of men of this height and age who weigh between 180 lb. and 195 lb., whose excess, that is to say, is between 20 per cent. and 30 per cent., will supply 112 actual, as against 100 expected, deaths, while as the disproportion increases the mortality steadily advances, a group of overweights to the extent of 30 per cent. and upwards yielding 130 actual for each 100 expected deaths. The age of overweights is, as we have said, a second factor of much importance, for the danger of excessive bulk advances with years. Whereas at the age of 22 an excess of weight surpassing 30 per cent. gives a mortality of 109 for each 100 expected deaths, the same excess of weight gives at the age of 35 years 130 deaths, and at 65 no fewer than 172. It appears, then, that a moderate degree of overweight is not harmful before the age of 30. "Beyond this age," says Dr. Symonds, "the mortality among overweights rises rapidly with the age and with the weight. This will happen in spite of the utmost care in examining and selecting the risks. Nothing seems to help these cases as a class. Even a long-lived ancestry only reduces the excessive mortality a little, but does not bring it within normal bounds." As with overweight, so with underweight, the disproportion is not seriously regarded until it reaches 20 per cent., but even in advanced degrees underweight appears to be a factor of relatively slight importance as regards longevity. Only when it is associated with a pronounced family tendency towards tuberculosis does it assume much consequence. For this reason, tuberculosis being essentially a disease of early life, underweight ceases to be of practical importance after the age of 35, though before this age the combination of underweight and a tuberculous family history raises the mortality to 180 for each 100 expected deaths.

Dr. Symonds concludes his communication with an interesting table showing the causes of death among the overweights and underweights at his office compared with the general experience of the same institution. The results are not altogether surprising, but serve to explain the dangers which attend the anomalies with which his paper deals. Overweight secures a marked degree of immunity from tuberculosis. Among the overweights there occurred less than one quarter of the amount of fatal tuberculosis met with in the general experience and less than one-sixth of that met with among the underweights. Pneumonia, also, contrary to the general belief, dealt less hardly with the overweights than with the common run of insured lives. But here the virtue of overweight ceases. The amount of diabetes it supplies is more than twice that of the general experience: arterial diseases, including cerebral hæmorrhages and congestions, are decidedly more frequent among the overweights than among ordinary individuals, and the same is true of organic diseases of the heart. Cirrhosis of the liver is three and a half times more frequent a cause of death among over-

213607

weights than among ordinary accepted lives, and Bright's disease nearly twice as prevalent. The evil prominence of underweight, on the other hand, is limited to respiratory diseases. In these classes the underweights supply many more deaths than the general experience, and since the deaths are for the most part early, this peculiar susceptibility of underweights is of serious import from the assurance point of view. But in almost all other classes the mortality of underweights compares favourably with the general experience. Diabetes and cirrhosis of the liver are only half as common as causes of death among underweights as among the bulk of assured lives: organic disease of the heart and Bright's disease are decidedly less frequent. Finally, no overweight is reported as having died of senility, while 27 such deaths are credited to underweights. More than this, no overweight attained the age of 80 years, while of the underweights 44 passed this age and two lived to be older than 90 years.

